# Single-Cell Profiling of Kidney Transplant Recipients With Immunosuppressive Treatment Reveals the Dynamic Immune Characteristics

**DOI:** 10.3389/fimmu.2021.639942

**Published:** 2021-04-20

**Authors:** Yongguang Liu, Xiaoyou Liu, Song Zhou, Ruiquan Xu, Jianmin Hu, Guorong Liao, Jun Liao, Zefeng Guo, Yuzhu Li, Siqiang Yang, Shichao Li, Hua Chen, Ying Guo, Min Li, Lipei Fan, Liuyang Li, Ming Zhao, Ding Liu

**Affiliations:** ^1^ Department of Organ Transplantation, Zhujiang Hospital, Southern Medical University, Guangzhou, China; ^2^ Department of Organ Transplantation, The First Affiliated Hospital of Guangzhou Medical University, Guangzhou, China; ^3^ Department of Urology, First Affiliated Hospital of Gannan Medical University, Ganzhou, China

**Keywords:** kidney transplant recipients, CyTOF, single-cell profiling, immune characteristics, immunosuppressive treatment

## Abstract

Kidney transplantation is currently the first choice of treatment for various types of end-stage renal failure, but there are major limitations in the application of immunosuppressive protocols after kidney transplantation. When the dose of immunosuppressant is too low, graft rejection occurs easily, while a dose that is too high can lead to graft loss. Therefore, it is very important to explore the immune status of patients receiving immunosuppressive agents after kidney transplantation. To compare the immune status of the recipient’s whole peripheral blood before and after receipt of immunosuppressive agents, we used single-cell cytometry by time-of-flight (CyTOF) to detect the peripheral blood immune cells in five kidney transplant recipients (KTRs) from the Department of Organ Transplantation of Zhujiang Hospital of Southern Medical University before and after receiving immunosuppressive agents. Based on CyTOF analysis, we detected 363,342 live single immune cells. We found that the immune cell types of the KTRs before and after receipt of immunosuppressive agents were mainly divided into CD4+ T cells, CD8+ T cells, B cells, NK cells/γδ T cells, monocytes/macrophages, granulocytes, and dendritic cells (DCs). After further reclustering of the above cell types, it was found that the immune cell subclusters in the peripheral blood of patients underwent major changes after receipt of immunosuppressants. After receiving immunosuppressive therapy, the peripheral blood of KTRs had significantly increased levels of CD57+NK cells and significantly decreased levels of central memory CD4+ T cells, follicular helper CD4+ T cells, effector CD8+ T cells, effector memory CD8+ T cells and naive CD8+ T cells. This study used CyTOF to classify immune cells in the peripheral blood of KTRs before and after immunosuppressive treatment, further compared differences in the proportions of the main immune cell types and immune cell subgroups before and after receipt of immunosuppressants, and provided relatively accurate information for assessment and treatment strategies for KTRs.

## Introduction

Kidney transplantation is currently recognized by the international medical community as the first choice of treatment for various types of end-stage renal failure (1, 2). Optimized immunosuppressive regimens and new immune detection technologies have significantly improved the short-term outcomes of transplant recipients after surgery, but effective methods for immune system function monitoring in patients after kidney transplantation are still lacking, leading to many blind spots in the clinical application of immunosuppressive agents. When the dose of immunosuppressant is too low, graft rejection can easily occur, and improper handling can lead to loss of the graft. In contrast, too high a dose of immunosuppressant can impair the patient’s immune system, making them prone to bacterial infection. Furthermore, infections with fungi, viruses, and protozoa have become the most important causes of death in transplant patients (1–3). Therefore, it is important to explore the immune status of patients receiving immunosuppressive agents after kidney transplantation.

The most critical role of the human immune system is to effectively recognize and eliminate foreign antigens while protecting the normal tissue structure from damage. The immune system has a protective effect on self-antigens; that is, there is a mechanism of self-tolerance. A healthy immune system has a variety of central and peripheral tolerance mechanisms, such as clonal loss, clonal function inhibition, immune escape, and immune exemption, which play vital roles in inhibiting the activation of autoreactive immune cells (4, 5). When infection, inflammation, or immune rejection occurs, the proportion and distribution of immune cells also change. Therefore, detecting changes in the immune microenvironment in the peripheral blood of KTRs after the application of immunosuppressive agents can aid in evaluating immune status (4). For example, the proportion of regulatory T cells (Tregs) in the peripheral blood of immune-tolerant KTRs was found to be increased (6, 7). When comparing KTRs with stable renal function and KTRs with chronic rejection, the numbers of CD19+CD24highCD27+ B10 (Breg) cells and CD19+ CD24highCD38high transitional B cells producing IL-10 in the peripheral blood were significantly increased in the tolerant KTRs (8). Therefore, exploring immune cell profiles in the peripheral blood of KTRs before and after receipt of immunosuppressive therapy is helpful to assess their immune status and guide clinical diagnosis and treatment.

As a representative application of single-cell analysis, CyTOF uses metal isotope-labeled antibodies to overcome the limitations of emission spectrum signal overlaps among traditional flow channels and can simultaneously detect as many as 40 parameters in a single cell to achieve accurate analysis of cell subpopulations (9). CyTOF has played an important role in the immune profiling of various diseases, such as ischemic stroke (10) and lacrimal glands (11). Therefore, this study used CyTOF to explore differences in the immunological profiles before and after immunosuppressive treatment in KTRs to further provide accurate assessment and treatment strategies.

## Methods

### Clinical Samples

We collected KTRs at Zhujiang Hospital of Southern Medical University between July 2020 and August 2020 and collected matched peripheral blood samples before and after immunosuppressive therapy after kidney transplantation. In total, 5 patients were included in this study, and the clinical data are included in **Table S1**. The immunosuppressive protocols of the recipients were described in a previous study and are detailed in the supplementary methods (12). These patients were followed up for one month after receiving immunosuppressive therapy, and none of them showed any progress. This study was approved by the Ethics Committee of Zhujiang Hospital of Southern Medical University, and all patients provided informed consent.

### CyTOF Analysis

CyTOF analysis was performed according to a previously described protocol (13). CyTOF analysis was used to detect immune cells in peripheral blood samples, and 40 immune cell-related markers were included in this analysis (**Table S2**).

### Raw Data Preprocessing and Cluster Analysis

MATLAB was used to normalize the preprocessing of.fcs files (14), remove the influence of noise from batches, and obtain the effective data of live single immune cells for the next analysis. Cytobank (15) (https://www.cytobank.org/) was used to analyze.fcs file data. vi-SNE plots were generated by the t-distribution random neighborhood embedding (t-SNE) algorithm, and CD45+ cells were clustered to analyze the differences in phenotype and relative content of different cell subpopulations. In addition, the SPADE algorithm (16) was used to analyze the expression patterns of immune cells in different groups and classify these cells by hierarchical clustering.

### Statistical Method

According to the characteristics of the data distribution, a paired Mann–Whitney U test was applied to compare differences between various immune cell subgroups in the peripheral blood of KTRs before and after immunosuppressive treatment, and the data were analyzed using R software. A two-sided P <0.05 was considered statistically significant.

## Results

### Immune Cells in the Peripheral Blood of KTRs

**Figure 1:**
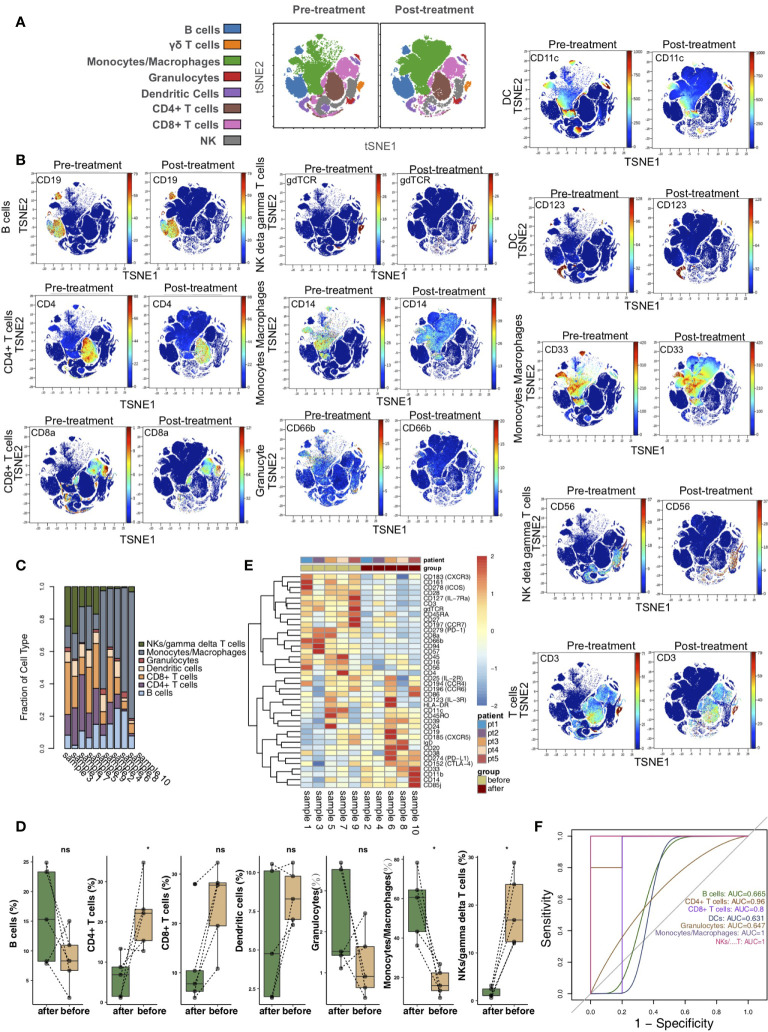
The immune landscape of the kidney. **(A)** t-SNE plots of CD4+ T cells, CD8+ T cells, B cells, NK cells/γδ T cells, monocytes/macrophages, granulocytes, and dendritic cells. **(B)** t-SNE plots of CD19, CD3, CD4, CD8a, CD56, gdTCR, CD33, CD14, CD66b, CD11c, and CD123 expression in the pre- and posttreatment groups. **(C)** The proportions of main cell types in all samples. **(D)** Comparison of the proportions of main cell types between the pre- and posttreatment groups. **(E)** Heatmap of the marker expression for all samples. **(F)** ROC curve of the main cell types predicting the pre- and posttreatment groups. *P<0.05; ns, not significant.

According to the immune cell markers, CD45+ cells were divided using the manual gated circle function of the Cytobank platform (**Figure S1**). vi-SNE is a bioinformatic analysis method based on t-SNE. This method can convert multidimensional data into single-cell two-dimensional visualization data and is widely used in mass spectrometry flow data analysis. vi-SNE analysis of CD45+ cells was performed with various markers, such as those for CD4+ T cells (marker: CD4), CD8+ T cells (marker: CD8a), B cells (marker: CD19), NK cells/γδ T cells (markers: CD56 and gdTCR), monocytes/macrophages (markers: CD33 and CD14), granulocytes (marker: CD66b), and dendritic cells (DCs; markers: CD11c and CD123) (**Figure 1A**). We visualized the expression of the above markers in the pretreatment and posttreatment groups (**Figure 1B**). The proportions of the 7 immune cell types in each sample were different (**Figure 1C**). Next, we compared the relative abundances of immune cells in the peripheral blood of KTRs between the pretreatment and posttreatment groups (**Figure 1D**) and found that, compared with the pretreatment group, the relative abundance of CD4+ T cells in the posttreatment group was significantly downregulated (P <0.05). In contrast, the relative content of monocytes/macrophages in the posttreatment group was significantly higher than that in the pretreatment group (P <0.05). Additionally, the expression of immune cell markers in the peripheral blood of patients was different between the pretreatment and posttreatment groups (**Figure 1E** and **Table S1**). ROC analysis showed that the application of CD4+ T cells, monocytes/macrophages or NK cells/γδ T cells was the strongest for predicting the efficacy of immunosuppressive agents in the pretreatment and posttreatment groups (AUC = 0.96, 1 and 1, respectively; **Figure 1F**).

### Differences in CD4+ T Cells Between Pretreatment and Posttreatment

**Figure 2:**
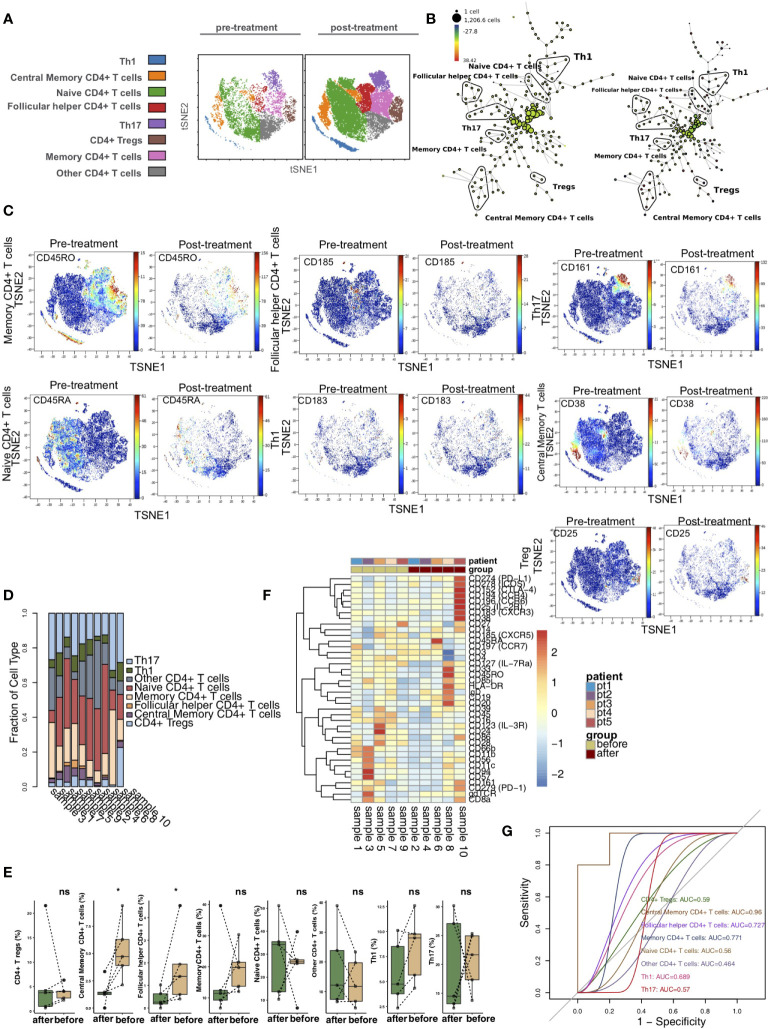
CD4+ T cell subclusters in the kidney. **(A)** t-SNE plots of central memory CD4+ T cells, follicular helper CD4+ T cells, naive CD4+ Tregs, Th1 cells and Th17 cells. **(B)** In SPADE trees, node color is scaled to the fold change in CD38 content, and node size is scaled to the number of cells. **(C)** t-SNE plots of CD38, CD185, CD45RA, CD25, CD183 and CD161 expression in the pre- and posttreatment groups. **(D)** The proportions of CD4+ T cell subclusters in all samples. **(E)** Comparison of the proportions of CD4+ T cell subclusters between the pre- and posttreatment groups. **(F)** Heatmap of the marker expression of CD4+ T cells for all samples. **(G)** ROC curve of CD4+ T cell subclusters predicting the pre- and posttreatment groups. *P < 0.05; ns, not significant.

To explore the differences in CD4+ T cells between pretreatment and posttreatment in the KTRs, we further reclustered the CD4+ T cells. Based on previous studies and the differences in the expression of markers on CD4+ T cells (17–20), we used vi-SNE to recluster CD4+ T cells and further divided them cells into 8 immune cell subgroups (**Figure 2A**), including central memory CD4+ T cells (marker: CD38), follicular helper CD4+ T cells (Tfh cells; marker: CD185), naive CD4+ T cells (marker: CD45RA), CD4+ Tregs (marker: CD25), Th1 CD4+ T cells (marker: CD183), Th17 CD4+ T cells (marker: CD161), memory CD4+ T cells (marker: CD45RO) and other CD4+ T cells. We used the SPADE algorithm to perform hierarchical clustering of CD4+ T cells based on marker expression (**Figure 2B**). Next, we visualized the expression levels of the above markers in the pretreatment and posttreatment groups (**Figure 2C**). The relative content of CD4+ T cell subsets in each sample was diverse (**Figure 2D**). Next, we compared the difference in the content of CD4+ T cell subsets in the peripheral blood of patients between the pretreatment and posttreatment groups (**Figure 2E**) and found that, compared with the pretreatment group, the central memory CD4+ T cell and follicular helper CD4+ T cell levels in the posttreatment group were significantly downregulated (P <0.05). In addition, we found that the expression patterns of CD4+ T cell subsets in the peripheral blood of patients greatly differed between pretreatment and posttreatment (**Figure 2F** and **Table S2**). ROC analysis indicated that central memory CD4+ T cells best predicted the pretreatment and posttreatment groups (AUC = 0.96; **Figure 2G**).

### Differences in CD8+ T Cells Between Pretreatment and Posttreatment

**Figure 3:**
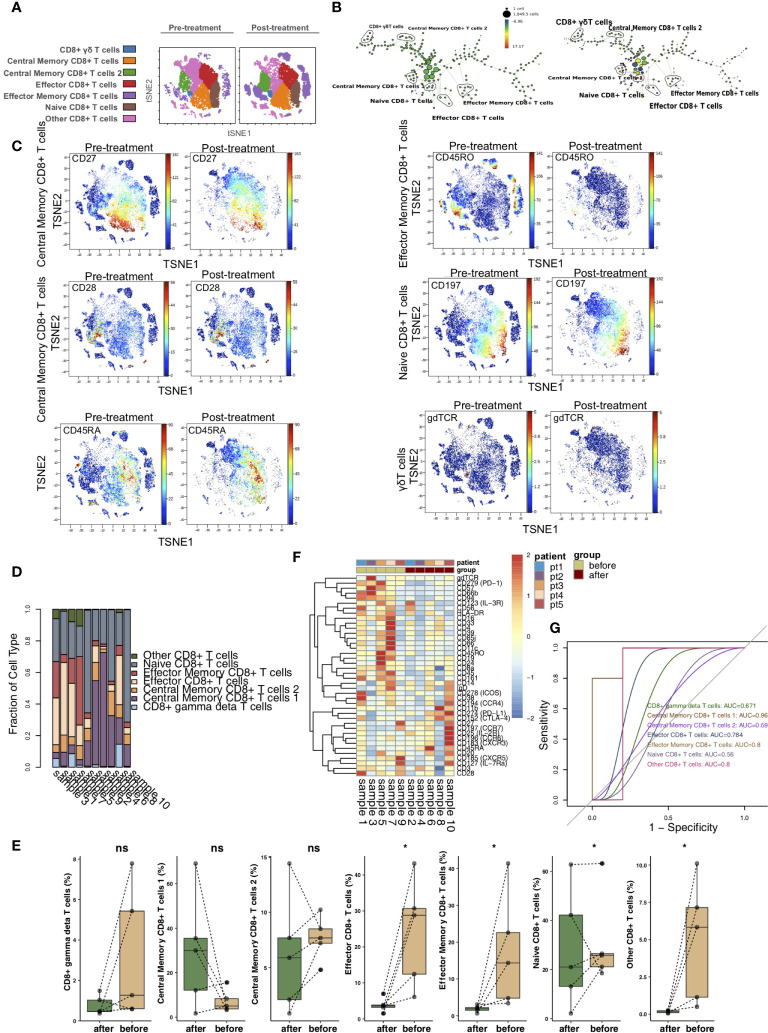
CD8+ T cell subclusters in the kidney. **(A)** t-SNE plots of CD8+ γδ T cells, central memory CD8+ T cells 1, central memory CD8+ T cells 2, naive CD8+ T cells, effector CD8+ T cells, effector memory CD8+ T cells and other CD8+ T cells. **(B)** In SPADE trees, node color is scaled to the fold change in CD197 (CCR7) content, and node size is scaled to the number of cells. **(C)** t-SNE plots of gdTCR, CD27, CCR7, CD28, CD45RA and CD45RO expression in the pre- and posttreatment groups. **(D)** The proportions of CD8+ T cell subclusters in all samples. **(E)** Comparison of the proportions of CD8+ T cell subclusters between the pre- and posttreatment groups. **(F)** Heatmap of the marker expression of CD8+ T cells for all samples. **(G)** ROC curve of CD8+ T cell subclusters predicting the pre- and posttreatment groups. *P < 0.05; ns, not significant.

To illustrate the differences in CD8+ T cells before and after treatment with immunosuppressive agents in the KTRs, we further re-clustered CD8+ T cells. CD8+ T cells were further divided into 7 immune cell subgroups (**Figure 3A**), including CD8+ γδ T cells (marker: gdTCR), central memory CD8+ T cells 1 (markers: CD27high and CCR7low), central memory CD8+ T cells 2 (marker: CD28), naive CD8+ T cells (markers: CCR7high and CD45RA), effector CD8+ T cells (markers: CCR7low, CD27low, and CD45RA), effector memory CD8+ T cells (marker: CD45RO) and other CD8+ T cells. The SPADE algorithm was used to perform hierarchical clustering of CD8+ T cells according to the expression of each marker (**Figure 3B**). **Figure 3C** shows the expression of markers in the pretreatment and posttreatment groups. The proportion of CD8+ T cell subsets between KTRs was different (**Figure 3D**). Additionally, we found that, compared with those in the pretreatment group, the level of effector CD8+ T cells and the relative abundances of effector memory CD8+ T cells and naive CD8+ T cells in the posttreatment group were significantly downregulated (P <0.05; **Figure 3E**). We discovered that the expression patterns of CD8+ T cell subsets in the peripheral blood of patients before and after treatment were significantly different, including PD-L1, CTLA-4, CCR7, CCR6, CXCR5, IL-7Ra and IL-2R (**Figure 3F** and **Table S3**). ROC analysis showed that central memory CD8+ T cells 1 and effector memory CD8+ T cells were best able to predict the immunosuppressive agents before and after treatment (AUC = 0.96 and 0.8, respectively; **Figure 3G**).

### Differences in NK Cells/γδ T Cells Between Pretreatment and Posttreatment

**Figure 4:**
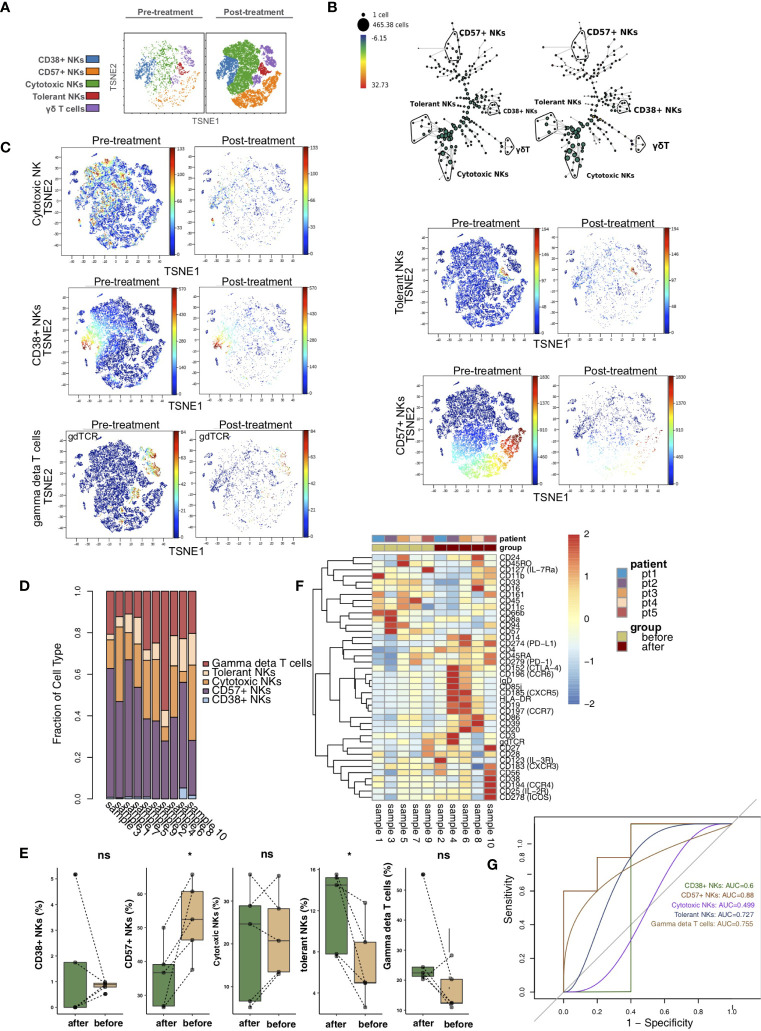
NK cell/γδ T cell subclusters in the kidney. **(A)** t-SNE plots of CD38+ NK cells, CD57+ NK cells, cytotoxic NK cells, tolerant NK cells and γδ T cells. **(B)** In SPADE trees, node color is scaled to the fold change in gdTCR content, and node size is scaled to the number of cells. **(C)** t-SNE plots of gdTCR, CD38, CD57, CD56 and CD11b expression in the pre- and posttreatment groups. **(D)** The proportions of NK cell/γδ T cell subclusters in all samples. **(E)** Comparison of the proportion of NK cell/γδ T cell subclusters between the pre- and posttreatment groups. **(F)** Heatmap of the marker expression of NK cells/γδ T cells for all samples. **(G)** ROC curve of NK cell/γδ T cell subclusters predicting the pre- and posttreatment groups. *P < 0.05; ns, not significant.

vi-SNE analysis showed that NK cells/γδ T cells were divided into CD38+ NK cells (markers: CD38bright and CD56dim), CD57+ NK cells (marker: CD57), cytotoxic NK cells (markers: CD11bbright and CD56dim), tolerant NK cells (marker: CD56bright) and γδ T cells (marker: gdTCR) according to specific markers (**Figure 4A**) (21–25). **Figure 4B** shows the hierarchical clustering of NK cells/γδ T cells according to the expression of each marker using the SPADE algorithm. We visualized the expression levels of the above unique markers in the pretreatment and posttreatment groups (**Figure 4C**). The relative content of NK cells/γδ T subsets in each sample was different (**Figure 4D**). We found that CD57+ NK cells were significantly downregulated in the posttreatment group compared with the pretreatment group (P <0.05). In contrast, the content of tolerant NK cells was significantly increased after treatment (P <0.05; **Figure 4E**). In addition, the heat map shows that the expression patterns of NK cell/γδ T cell subsets in the peripheral blood of patients before and after treatment were different, such as CCR6, CXCR5, CCR7, CXCR3, CCR4, IL-2R and IL-4R (**Figure 4F**; **Table S4**). ROC analysis showed that CD57+ NK cells best predicted the efficacy of immunosuppressive agents before and after treatment (AUC = 0.88, **Figure 4G**).

### Differences in DCs Between Pretreatment and Posttreatment

**Figure 5:**
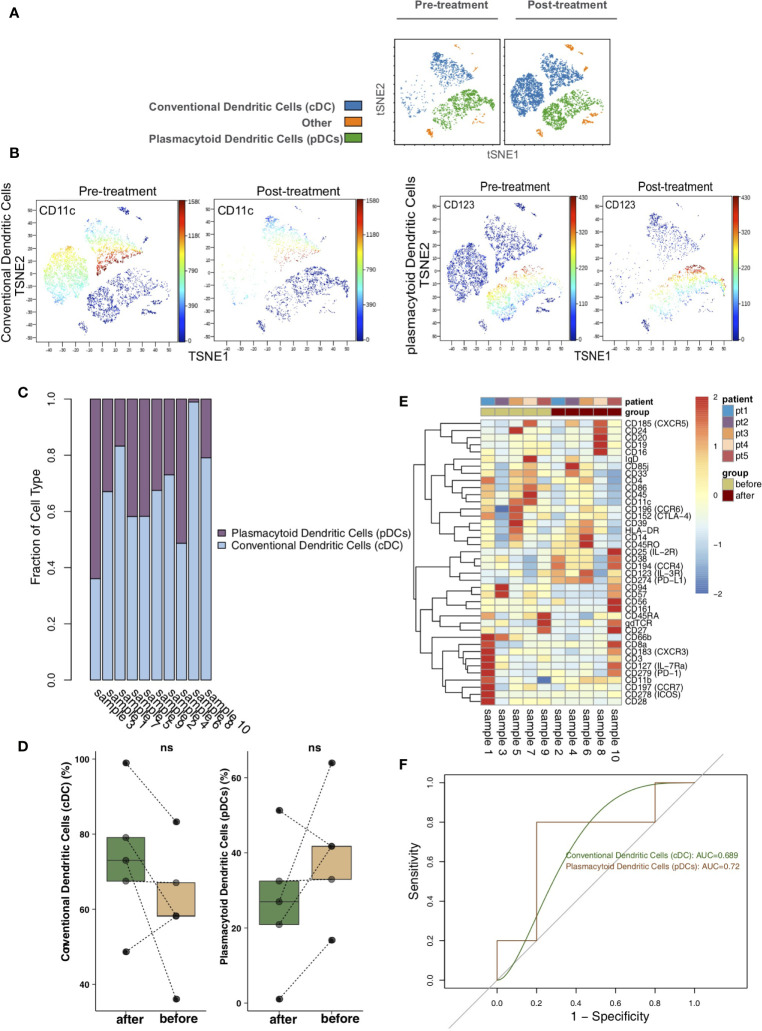
Dendritic cell (DC) subclusters in the kidney. **(A)** t-SNE plots of conventional dendritic cells (cDCs) and plasmacytoid dendritic cells (pDCs). **(B)** t-SNE plots of CD11c and CD123 expression in the pre- and posttreatment groups. **(C)** The proportions of DC subclusters in all samples. **(D)** Comparison of the proportion of DC subclusters between the pre- and posttreatment groups. **(E)** Heatmap of the marker expression of DCs for all samples. **(F)** ROC curve of DC subclusters predicting the pre- and posttreatment groups. ns, not significant.

To analyze the differences in DCs before and after treatment with immunosuppressive agents in the KTRs, DCs were re-clustered into 2 cell subgroups (**Figure 5A**), including conventional dendritic cells (cDCs; marker: CD11c) and plasmacytoid dendritic cells (pDCs; marker: CD123) using vi-SNE (**Figure 5B**). The content of DC subpopulations in each sample was different (**Figure 5C**). Next, we compared the relative abundances of DC subgroups in the peripheral blood of patients before and after treatment (**Figure 5D**) and found that there were no significant changes in DC subgroups in the peripheral blood of patients before and after treatment. The expression patterns of DC subgroups in the peripheral blood of patients before and after treatment were also different (**Figure 5E**; **Table S5**). ROC analysis showed that pDCs strongly predicted the efficacy of immunosuppressive agents before and after treatment (AUC = 0.72, **Figure 5F**).

### Differences in B Cells Between Pretreatment and Posttreatment

**Figure 6:**
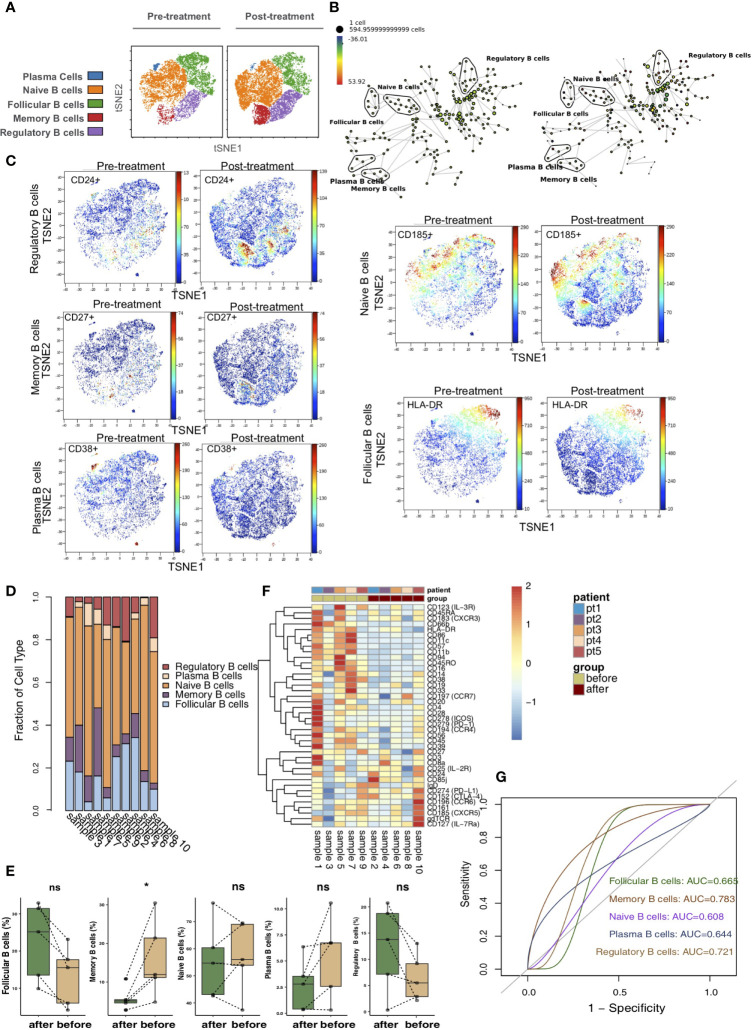
B cells subclusters in the kidney. **(A)** t-SNE plots of follicular B cells, memory B cells, naive B cells, plasma B cells and regulatory B cells. **(B)** In SPADE trees, node color is scaled to the fold change in CD185 (CCR5) content, and node size is scaled to the number of cells. **(C)** t-SNE plots of CD20, CD27, CD185 and CD38 expression in the pre- and posttreatment groups. **(D)** The proportions of B cell subclusters in all samples. **(E)** Comparison of the proportion of B cell subclusters between the pre- and posttreatment groups. **(F)** Heatmap of the marker expression of B cells for all samples. **(G)** ROC curve of B cell subclusters predicting the pre- and posttreatment groups. *P < 0.05; ns, not significant.

According to differences in the expression of B cell markers, we used vi-SNE to recluster B cells and further manually divided them into 5 cell subgroups (**Figure 6A**), including follicular B cells (markers: CD20 and HLA-DR), memory B cells (marker: CD27), naive B cells (marker: CD185), plasma B cells (markers: CD38) and regulatory B cells (marker: CD24) according to specific markers. The SPADE algorithm was applied to perform hierarchical clustering of B cells based on the expression of markers (**Figure 6B**). Then, we visualized the expression of the above immune cell markers in the pretreatment and posttreatment groups (**Figure 6C**). The bar plot shows that KTRs harbored relatively different proportions of B cell subgroups (**Figure 6D**). Next, we compared the relative abundances of B cell subpopulations in the peripheral blood of patients between the pretreatment and posttreatment groups (**Figure 6E**) and found that, compared with the posttreatment group, the peripheral blood of the pretreatment group had an increased level of memory B cells (P <0.05). The expression patterns of B cell subsets in the peripheral blood of patients before and after treatment are shown in **Figure 6F** (**Table S6**). ROC analysis showed that memory B cells and regulatory B cells best predicted the efficacy of immunosuppressive agents before and after treatment (AUC = 0.783 and 0.721, **Figure 6G**).

## Discussion

Related to the long-term functional and stable survival of grafts after kidney transplantation, reducing or removing immunosuppressive agents as much as possible and inducing transplant tolerance are research hotspots in the field of kidney transplantation, and they are also the goals of transplant doctors. Although the short-term survival rate after kidney transplantation has improved significantly in recent years, long-term survival remains a problem in the transplant community. Studies have reported that one of the main factors of kidney graft loss is the body’s chronic rejection of allogeneic antigens (26, 27). However, although the use of immunosuppressive agents has largely suppressed the occurrence of rejection, immunosuppressants may also contribute to subsequent renal rejection (28, 29). Additionally, some recipients in solid organ transplantation (non-kidney) experience renal failure due to the toxicity of immunosuppressive agents (30). Therefore, evaluating the immune status of recipients after renal transplantation before and after receipt of immunosuppressive therapy is of guiding significance for evaluating whether a KTR needs to tolerate or completely discontinue the immunosuppressive agent or in determining if there is rejection that requires further clinical treatment. In this study, CyTOF analysis was used to further explore the immune atlas before and after immunotherapy after kidney transplantation at single-cell resolution.

T cells can be divided into CD4+ T cells, CD8+ T cells and Treg cells according to their function (2, 31). Th cells and cytotoxic T lymphocytes (CTLs) play important roles in the immune responses involved in human infection, inflammation, elimination of pathogens, and rejection of transplanted organs. Increasing evidence indicates that multiple effector CD4+ T cell subsets play roles in xenograft rejection (2, 32). In addition to directly reducing activity through CTLs, xenograft rejection can be achieved through T cell-mediated mechanisms, including the production of cytokines, the aggregation and activation of cytotoxic cells, and the production of xenograft antibodies from B cells (2, 33). Follicular helper T cells (Tfh) are a recently discovered CD4+ helper T lymphocyte subset that is different from previously defined Th cell subsets, such as Th1/Th2, Th17, Treg and Th9 cells (31). These cells express the chemokine receptor CXCR5, PD1 and inducible costimulatory molecules (ICOS); are mainly active in secondary lymphoid organs; and can also be present in tertiary lymphatic structures of the transplanted kidney (34). A study found that the number of Tfhs in KTRs remained stable after transplantation, while the ability to express IL-21 decreased under immunosuppression (35). Other studies in renal transplant recipients have demonstrated increased Tfh cell numbers before the development of donor-specific antibodies (DSA), in association with antibody-mediated rejection (ABMR) or with the development of anti-HLA antibodies (36–38). Furthermore, anti-rejection therapy with alemtuzumab significantly lowers the number of Tfh cells in kidney transplant recipients and contributes to the stable status of the kidney transplant (39). A study compared the proportion and function of follicular helper T cells in the blood between patients with operative tolerance and patients with stable graft function. It was found that the proportion of follicular helper T cells in patients with tolerance was decreased, and their function was impaired upon coculture with B cells (36). CD8+ T lymphocytes can directly attack inhibitors and eliminate target cells through cytotoxicity. In this study, the proportion of CD4+ T cells in the peripheral blood of the posttreatment group was significantly lower than that of the pretreatment group. Subgroup analysis showed that the proportions of central memory CD4+ T cells and follicular helper CD4+ T cells in the peripheral blood of the posttreatment group were reduced compared with those in the pretreatment group.

CD8+ T lymphocytes can specifically recognize the transplanted kidney. They can pass through the basement membrane of the renal tubules, proliferate and induce apoptosis in renal tubular cells (2). We found that patients receiving immunosuppressive therapy after kidney transplantation had relatively few effector CD8+ T cells and effector memory CD8+ T cells in their peripheral blood. Therefore, the relative proportions of some CD8+ T cell subgroups (such as effector CD8+ T cells and effector memory CD8+ T cells) decreased after receipt of immunosuppressive agents, maintaining the recipient’s immune tolerance. Additionally, NK cells have antitumor, antiviral, and anti-transplanted organ activities and participate in the regulation of T and B lymphocytes and their immunoregulatory functions. NK cells maintain balance and exert killing effects through their receptors (40–42). Vacher-Coponat et al. (43) reported that the combined application of multiple immunosuppressants had a significant inhibitory effect on NK cells and that the main effect of tacrolimus on the immune system is to inhibit the activation and proliferation of T cells. Studies have shown that tacrolimus can also affect the proliferation and function of NK and NKT cells (44). Recently, many studies have shown that tolerant NK cell populations (45) are related to graft immune tolerance (42, 46, 47). We found that the proportion of tolerant NK cells in the peripheral blood in the posttreatment group was significantly higher than that in the pretreatment group, while the content of CD57+ NK cells in the peripheral blood in the posttreatment group was higher than that in the pretreatment group.

B cells are important immune cells in the human immune response. In addition to secreting specific antibodies, B cells can participate in immune responses through antigen presentation, costimulation, and cytokine secretion (48). Additionally, there is a class of B cells that has immunosuppressive effects, called Bregs (49). Bregs can secrete inhibitory cytokines, such as IL-35 and IL-10, and express membrane surface molecules, such as FasL and CD1d, to exert immunosuppressive effects. They can also inhibit Th cells, CTLs, DCs, macrophages and other immune cells involved in the development of various immune conditions, such as autoimmune diseases and responses to infection, tumors, and organ transplantation (50–52). Additionally, MHC molecules from the donor can activate Bregs, further inhibit T cell-mediated rejection, and exert an immunosuppressive effect in the MHC mismatch area, thereby prolonging the survival time of MHC mismatched cells during the allogeneic suppression of the immune response to promote Treg expansion (53, 54). Several studies have shown that, compared with those in the peripheral blood of chronic rejection recipients, Breg levels in the peripheral blood of tolerant KTRs are significantly increased (55, 56). Given the immunosuppressive function of Bregs, studies have shown that targeting Bregs may become a new immunosuppressive treatment in the field of transplantation (57, 58). Coquet reported that the adoptive transfer of Bregs to chronic collagen arthritis mice inhibited Th1 differentiation, reduced the severity of arthritis, and promoted the resolution of the disease (59). In the transplant tolerance model, splenic B cells of long-term survival animals after transplantation were purified and then adoptively transferred to the recipient animal. It was found that the Tregs in the recipient’s spleen rapidly expanded; at the same time, the CD4+CD25-T cells in the body interacted with the Bregs (60). This study found that the abundance of Bregs in the peripheral blood in the posttreatment group was higher than that in the pretreatment group. However, due to the limited sample size, this difference was not statistically significant.

This study had the following limitations: 1) The sample size was small, and a larger independent cohort could enable future analysis of more cell subpopulations in KTRs to clarify the immune status and treatment strategies after kidney transplantation; 2) This study lacked immunohistochemical verification of kidney tissue samples from KTRs; 3) There was strong heterogeneity in the immune status of the peripheral blood among different KTRs. We hope that more peripheral blood samples collected from KTRs before and after immunosuppressive therapy can be included in the future to explore the effect of heterogeneity among individuals on immune status.

## Conclusions

In this study, CyTOF was used to classify immune cells in the peripheral blood of KTRs before and after receipt of immunosuppressive agents, and differences in the proportions of the main immune cells and immune cell subgroups were further compared between pretreatment and posttreatment groups. We found that the abundances of activated immune cell subsets (such as central memory CD4+ T cells, follicular helper CD4+ T cells, effector CD8+ T cells and effector memory CD8+ T cells) in the peripheral blood of patients after receipt of immunosuppressive therapy and the proportion of tolerant immune cells (such as tolerant NK cells) were increased in the posttreatment group. We hope that we can analyze more immune cell subsets in KTRs through analysis of a larger independent cohort in the future and that the data from these additional KTRs can help produce more accurate assessment and treatment strategies.

## Data Availability Statement

The original contributions presented in the study are included in the article/**Supplementary Material**. Further inquiries can be directed to the corresponding authors.

## Ethics Statement

The studies involving human participants were reviewed and approved by Zhujiang Hospital of The Southern Medical University. The patients/participants provided their written informed consent to participate in this study.

## Author Contributions

Conceptualization: YgL and DL. Formal analysis, YgL, XL, SZ and RX. Visualization: YgL, RX, XL and SZ. Writing–original draft, YgL, XL, RX and SZ. Writing–review & editing, JH, GL, JL, ZG, YzL, SY, SL, HC, YG, ML, LF, LL and MZ. All authors read and approved the final manuscript.

## Conflict of Interest

The authors declare that the research was conducted in the absence of any commercial or financial relationships that could be construed as a potential conflict of interest.

## Supplementary Material

Supplementary Figure 1 CD45+ CD66b- blood cells were manually selected and subjected to sequential gating to identify the neutrophil subpopulations with CyTOF.

Supplementary Table 1 The expression of immune cell markers in the peripheral blood of patients.

Supplementary Table 2 The expression of immune cell markers of CD4+ T cells of patients.

Supplementary Table 3 The expression of immune cell markers of CD8+ T cells of patients.

Supplementary Table 4 The expression of immune cell markers of NK cell/γδ T cell of patients.

Supplementary Table 5 The expression of immune cell markers of DCs of patients.

Supplementary Table 6 The expression of immune cell markers of B cells of patients.
