# Supplementary figures and images for: Single-Cell Profiling of Kidney Transplant Recipients With Immunosuppressive Treatment Reveals the Dynamic Immune Characteristics
Source: Front Immunol. 2021 Apr 20;12:639942. doi: 10.3389/fimmu.2021.639942 (PMC8093626; doi:10.3389/fimmu.2021.639942)

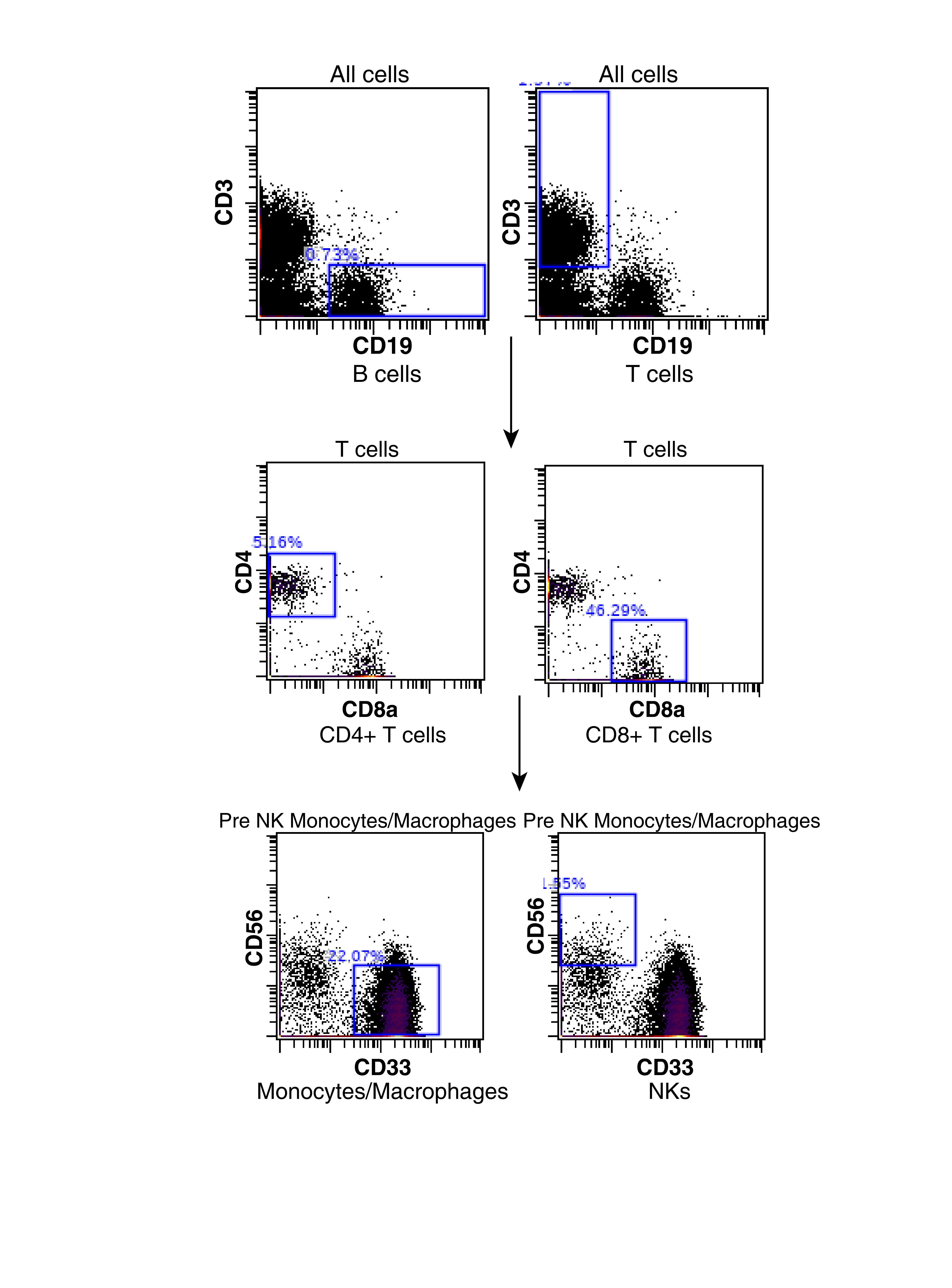

Supplement: Supplementary Figure 1 — CD45+ CD66b- blood cells were manually selected and subjected to sequential gating to identify the neutrophil subpopulations with CyTOF. [file Image_1.jpeg]
