# Supplementary material for: Single-Cell Profiling of Kidney Transplant Recipients With Immunosuppressive Treatment Reveals the Dynamic Immune Characteristics
Source: Front Immunol. 2021 Apr 20;12:639942. doi: 10.3389/fimmu.2021.639942 (PMC8093626; doi:10.3389/fimmu.2021.639942)
Supplement: Supplementary file 8 [file DataSheet_1.docx]

**Supplementary Methods**

**Patient blood collection**

Blood samples from patients who had end-stage renal disease after kidney transplantation were collected in EDTA-coated tubes at the Department of Organ Transplantation of Zhujiang Hospital of Southern Medical University. Concurrently, blood from corresponding patients who were treated with immunosuppressive agents was collected in EDTA-coated tubes. To ensure uniform treatment of the control and experimental materials, all donor blood samples were stored at 4°C overnight and then processed the next morning.

**Cell suspension generation from human WB**

Whole blood (WB) was subjected to red blood cell (RBC) lysis (RBC lysis buffer, eBioscience) twice at room temperature (RT) for 10 min. Cells were then washed with staining buffer (Dulbecco’s PBS+1% human serum+0.1% sodium azide+2 mM EDTA) and filtered through a 70-µm strainer. The cells were pelleted by centrifugation, and suspensions were prepared with gentle pipetting to reach a final concentration of 3×10^6^ cells per 100 µL of buffer.

**Mass cytometry antibodies**

Metal-conjugated antibodies were purchased directly from Fluidigm for available targets. For all other targets, purified antibodies were purchased as described previously.20 Antibody conjugates were prepared using the Maxpar Antibody Labeling Kit (Fluidigm) according to the manufacturer’s recommendations. Afterwards, the Maxpar-conjugated antibodies were stored in a PBS-based antibody stabilization solution (Candor Biosciences) supplemented with 0.05% sodium azide at 4°C. All antibodies were titrated before use.

**Mass cytometry (CyTOF)**

CyTOF was performed following previously described protocols[1]. For viability staining, cells were washed in PBS and stained with cisplatin (Fluidigm) at a final concentration of 5 µM. Prior to surface staining, RBC-lysed WB cells were resuspended in staining buffer for 15 min at RT to block Fc receptors. The surface antibody cocktail listed in Table S1 was added to the cell suspensions for 1 hour at 4°C. The cells were then washed with staining buffer and fixed with 1.6% paraformaldehyde (Thermo Fisher) for 15 min at RT. Afterwards, 1 mL of intercalation solution for each sample was prepared by adding Cell-ID Intercalator-Ir (Fluidigm) into Maxpar Fix and Perm Buffer (Fluidigm) to a final concentration of 125 nM (a 1:1,000 dilution of the 125 µM stock solution) and vortexing to mix. After fixation, the cells were resuspended in the intercalation solution and incubated overnight at 4°C. The cells were then washed in staining buffer and subsequently washed in Cell Acquisition Solution (CAS) (Fluidigm) to remove buffer salts. Next, the cells were resuspended in CAS with a 1:10 dilution of EQ Four Element Calibration beads (Fluidigm) and filtered through a 35-µm nylon mesh filter cap (Corning, Falcon). Samples were analyzed on a Helios 2 CyTOF Mass Cytometer (Fluidigm) equipped with a Super Sampler (Victorian Airship & Scientific Apparatus) at an event rate ≤500 events/s. Mass cytometry data files were normalized using the bead-based Normalizer[2] and were analyzed using Cytobank analysis software (https://www.cytobank.org/). For analysis of mass cytometry data with a self-organizing map (FlowSOM) in Cytobank[3], hierarchical clustering was used to identify seven metaclusters based on median marker expression (after arcsinh transformation with a cofactor equal to 5) from the visualization of t-distributed stochastic neighbor embedding (vi-SNE) results.

**Immunosuppressive regimens**

We converted twice-daily tacrolimus to once-daily tacrolimus on a 1:1 mg proportion basis for the total daily dose. Also, mycophenolate mofetil or enteric-coated mycophenolate sodium were converted to once-daily sirolimus 2 mg as starting dose. All the prescribed medicine was scheduled to be taken in the morning. The tacrolimus trough levels were measured, and the dose was adjusted to keep the trough level within the target range (3–8 ng/dL). In the same way, the sirolimus dose was adjusted to keep within the target range (3–8 ng/dL). All other drugs, including corticosteroids were changed to once-daily prescription or slow-release preparations if indicated. The drug levels were monitored by immunoassay methods in all centers.

Reference

1. Han G, Spitzer MH, Bendall SC, Fantl WJ, Nolan GP. Metal-isotope-tagged monoclonal antibodies for high-dimensional mass cytometry. Nat Protoc. 2018; 13: 2121–48.

2. Finck R, Simonds EF, Jager A, et al. Normalization of mass cytometry data with bead standards. Cytometry A. 2013; 83: 483–94.

3. Chen TJ, Kotecha N. Cytobank: providing an analytics platform for community cytometry data analysis and collaboration. Curr Top Microbiol Immunol. 2014; 377: 127–57.
